# Genomic analysis of non-carbapenem drug-resistant Gram-negative bacteria from advanced chronic liver disease (ACLD) patients suggests no evidence for in-house transmissions

**DOI:** 10.3389/fmicb.2025.1740272

**Published:** 2026-01-28

**Authors:** Christopher D. Best, Zahra Nemati, Tilman Schultze, Michael Hogardt, Camilla Cadoli, Marcus M. Mücke, Toska Wiedemann, Hans-Peter Erasmus, Christoph Welsch, Volkhard A. J. Kempf

**Affiliations:** 1 Institute for Medical Microbiology and Infection Control, University Hospital, Goethe University Frankfurt, Frankfurt am Main, Germany; 2 University Center for Infectious Diseases (UCI), University Hospital, Goethe University Frankfurt, Frankfurt am Main, Germany; 3 Landesbetrieb Hessisches Landeslabor, Giessen, Germany; 4 Medical Clinic 1, University Hospital, Goethe University Frankfurt, Frankfurt am Main, Germany

**Keywords:** advanced chronic liver disease, drug-resistant Gram-negative bacteria, infection control, molecular epidemiology, whole-genome sequencing

## Abstract

**Background:**

In advanced chronic liver disease (ACLD) patients, bacterial infections with multidrug-resistant Gram-negative bacteria (MDRGN) can progress to acute-on-chronic liver failure (ACLF) with high mortality rates. Particularly carbapenem-resistant Gram-negative bacteria (CR-GN) pose a significant threat due to limited antibiotic treatment options. However, non-carbapenem drug-resistant Gram-negative bacteria (NCR-DRGN) are clinically highly relevant, as they occur more frequently and may serve as precursors to CR-GN. This study aims to assess the prevalence, resistance mechanisms, and transmission dynamics of NCR-DRGN in ACLD patients including those with ACLF.

**Materials and methods:**

A prospective, single-center study was conducted at University Hospital Frankfurt. Over 32 months, ACLD patients were screened for NCR-DRGN by routine microbiology techniques. Whole-genome sequencing (WGS) of isolated bacteria was performed to analyze genetic diversity, resistance, and transmission patterns. Epidemiological links were explored through patient chart reviews.

**Results:**

NCR-DRGN were found in 12.1% (*n* = 22/182) of ACLD patients, comprising of 44 isolates, predominantly *Escherichia coli* (*n* = 40/44; 90.9%). All isolates were phenotypically classified as NCR-DRGN; however, one isolate was found to harbor a *bla*_OXA–244_ gene potentially affecting carbapenem treatment efficacy. Genomic analysis revealed significant diversity, with no evidence of clonal outbreaks, although one potential transmission event was identified.

**Conclusion:**

NCR-DRGN are prevalent in ACLD patients, with *E. coli* as the dominant pathogen. Standard hygiene measures appear effective in preventing transmission, emphasizing the importance of routine screening and infection control in this high-risk population.

## Introduction

Advanced chronic liver disease (ACLD) is frequently complicated by episodes of acute decompensation (AD), which significantly worsen the prognosis of liver patients. In a subset of patients, AD may progress to acute-on-chronic liver failure (ACLF), a severe clinical syndrome characterized by single or multiple organ failure and an alarming 3-month mortality of up to 51% (Moreau et al., 2013). Bacterial infections represent one of the most common precipitants of AD and are the leading etiology of ACLF (Trebicka et al., 2021). In this context, colonization with multidrug-resistant organisms (MDRO), particularly multidrug-resistant Gram-negative bacteria (MDRGN including NCR-DRGN) such as *Escherichia coli*, *Klebsiella pneumoniae*, and others, constitutes a substantial threat for patients with advanced chronic liver disease (ACLD) including those with ACLF.

The World Health Organization (WHO) has prioritized these pathogens to guide research and development efforts for new antibiotics (World Health Organization [WHO], 2024) as they limit therapeutic options and worsen patient outcomes by causing severe infections like bloodstream infections and spontaneous bacterial peritonitis. To date, liver transplantation (LT) remains the only curative intervention for ACLF (Arroyo et al., 2020). Active infections contraindicate transplantation, further highlighting the need for surveillance and prevention of MDRGN colonization (Adam et al., 2018). Effective strategies to reduce MDRGN transmission include implementation of robust screening protocols and rigorous hygiene measures (e.g., patient isolation, adherence to hygiene protocols). Early identification of MDRGN carriers through risk-based screening plays a vital role in mitigating transmission and improving patient outcomes. Therefore, avoiding MDRO transmission by preventive measures is essential for the management of ACLD patients.

Colonization with carbapenem-resistant MDRGN (CR-MDRGN) organisms is particularly concerning, as it is linked to highly increased mortality rates of ACLD patients (Ferstl et al., 2021). The mechanisms driving CR-MDRGN emergence are multifaceted and include: (i) combined porin loss and/or overexpression of β-lactam resistance genes, which regularly result from selective pressure due to extensive use of broad-spectrum antimicrobials in carbapenem-susceptible Gram-negative bacteria (Sommer et al., 2009; Meletis et al., 2014; Iredell et al., 2016); (ii) horizontal gene transfer of resistance determinants between bacterial species (Göttig et al., 2015); and (iii) direct interpatient transmission facilitated by shortcomings in infection control measures. While some centers report CR-MDRGN spread through dominant sequence types (STs) or carbapenemase production (Karampatakis et al., 2017; Macesic et al., 2018), data from the University Hospital Frankfurt (UHF) indicate that carbapenem resistance predominantly evolves from carbapenem-susceptible ancestors (Schultze et al., 2021). This finding underscores the importance for screening and implementing preventive measures against non-carbapenem-resistant drug resistant Gram negative bacteria (NCR-DRGN).

The herein presented study aims to assess the prevalence of NCR-DRGN in ACLD patients and to investigate mechanisms underlying antibiotic resistance. By combining conventional microbiological methods, whole-genome sequencing (WGS) and patient chart analysis, this research seeks to determine the epidemiological spread of NCR-DRGN in this vulnerable patient cohort.

## Materials and methods

### Patients and ethical approval

This study included a subgroup of 182 patients from the prospective longitudinal ACLF-I cohort study. The ACLF-I cohort comprises prospectively enrolled adult patients with ACLD who were hospitalized at the University Hospital Frankfurt (November 2020-June 2023). Eligibility was defined by any inpatient admission with established liver cirrhosis, while patients under 18 or over 80 years of age, with hepatocellular carcinoma beyond Milan criteria (Mazzaferro et al., 2011), any other malignant disease, ongoing immunosuppressive therapy, prior organ transplantation, or pregnancy were excluded.

All patients were subjected to screening measures for MDRGN (including NCR-DRGN) on admission and in the course of their hospital stay. These measures ground in nation-wide regulatory demands by the German Infection Protection Law (IfSG) and in-house guidelines outlined in the hygiene plan to prevent transmission of infective agents. Patients remained in the study until death or last follow-up. Study approval was obtained by the local Ethics Committee for Medical Research of the Medical Faculty, University Hospital, Goethe University, Frankfurt am Main, in accordance with the 1975 Declaration of Helsinki prior to research (file number 20-653). Informed consent was obtained upon study inclusion, and the database was pseudo-anonymized. Analysis of bacteria was also approved by the local Ethics Committee (waiver 2024-2145) and conducted within legal requirements given by German Infection Protection Law (IfSG), in particular §§13, 23 IfSG.

### Clinical laboratory procedures

All laboratory procedures were conducted in accordance with quality-controlled DIN ISO 15189:2024 standards (certificate number D–ML–13102–01–00). NCR-DRGN bacteria included in this study were defined as *Enterobacterales*, *Pseudomonas aeruginosa* and *Acinetobacter baumannii-calcoaceticus* complex having pheno- or genotypic resistance against at least third generation cephalosporins. Bacterial isolates with a phenotypic carbapenem resistance were excluded from further analysis. Rectal and nasopharyngeal swabs were the primary sample types utilized to screen for NCR-DRGN, with additional samples, including skin swabs, tracheal swabs, and urine samples as required. Amies collection and transport medium (Hain Lifescience, Nehren, Germany) was used for sample collection. For bacterial cultivation, all swabs were directly streaked onto CHROMagar™ ESBL plates (Mast Diagnostica, Paris, France) without prior enrichment culture allowing the growth and selection of ESBL-producing and other Gram-negative bacteria with resistance to penicillins and third generation cephalosporins.^1^ Species identification of suspected colonies was performed using matrix-assisted laser desorption ionization-time of flight (MALDI-TOF) analysis or the automated VITEK^®^ 2 system (bioMérieux, Nürtingen, Germany). Antibiotic susceptibility testing was carried out in accordance with the European Committee on Antimicrobial Susceptibility Testing (EUCAST) guidelines (EUCAST, 2020a,b). Methods included VITEK^®^ 2 automated systems, antibiotic gradient tests (Liofilchem, Roseto degli Abruzzi, Italy; Etest^®^, bioMérieux, Nürtingen, Germany), and agar diffusion assays (Oxoid, Wesel, Germany). Carbapenemase screening using the CARBA-5 immunochromatographic lateral flow assay (NG Biotech, Guipry, France) targeting NDM, VIM, IMP, OXA-48-like, and KPC carbapenemases was conducted based on the criteria outlined by the German National Antibiotic Susceptibility Committee (Hamprecht et al., 2021).

Definitions for colonization and infection were based on established microbiological and clinical standards: Colonization was defined as the detection of NCR-DRGN in nasal, rectal, or pharyngeal swabs. Infection was defined when NCR-DRGN were isolated from wounds or sterile compartments. According to German microbiological-infectiological quality (MiQ) standards (Schubert et al., 2020), urinary tract infection was confirmed in cases where no more than two bacterial pathogens, including NCR-DRGN or potential pathogens, were identified alongside leukocytes. In the absence of leukocytes, infection was defined by the presence of at least 10^4^ bacteria/mL of urine.

### Sequencing and bioinformatics analysis

Genomic DNA was extracted using DNeasy UltraClean Microbial Kit (Qiagen, Venlo, Netherlands) and sequenced on the Illumina NovaSeq X Plus platform (Illumina Inc., San Diego, USA) generating paired-end 150 bp reads (PE150). Raw reads were quality-trimmed, and Illumina-specific adapters were removed using Trimmomatic v0.39 (Bolger et al., 2014). Taxonomic classification of reads was performed as an additional quality control step using Kraken v2.1.3 (Wood et al., 2019). High-quality reads were assembled *de novo* into draft genomes using Unicycler v0.5.1 (Wick et al., 2017), and assembly quality was evaluated using QUAST v5.3.0 (Gurevich et al., 2013). Contigs shorter than 200 bp were discarded to ensure high assembly accuracy. Multi-locus sequence typing (MLST) was performed using mlst v2.23.0 (Seeman, 2019), and taxonomic classification of isolates was reconfirmed using Kraken v2.1.3 (Wood et al., 2019). Antibiotic resistance and virulence genes were identified by screening with ABRicate v1.0.1 (Seemann, 2016) and BLASTn searches against the ResFinder database (Zankari et al., 2012). For core genome based multilocus sequence typing (cgMLST) and minimum spanning tree construction of *E. coli* isolates, Ridom SeqSphere+ v10.5.0 was utilized applying the EnteroBase core genome MLST scheme.^2^ Genome annotation was performed using Prokka v1.14.6 (Seemann, 2014), and the resulting GFF files were used for pangenome analysis with Roary v3.13.0 (Page et al., 2015). The pangenome data were visualized using Roary2svg.pl.^3^ Prediction of localization of antimicrobial resistance (AMR) genes was based on RFPlasmid predictions.^4^ A tree based on the Roary alignment was calculated with IQTree2 version 2.2.5 using ModelFinder (Kalyaanamoorthy et al., 2017) to determine the best-fit model. Branch support was accessed by calculating 100,000 ultrafast bootstraps.

### Analysis of patient data records for epidemiological links

To identify potential epidemiological links in patients colonized or infected with NCR-DRGN bacteria, a review of patient data records was conducted. Clinical and microbiological data, including demographics, hospitalization history, and antimicrobial treatments, were analyzed to evaluate transmission dynamics and resistance patterns. The analysis focused on overlaps in time and location within healthcare facilities including (i) patient admissions and movements across general wards, intermediate care and intensive care units, (ii) close contacts located in the same room, (iii) visits to diagnostic and therapeutic units (e.g., endoscopy, ultrasonography, radiology, operating theaters) and (iv) visits to the outpatient clinics.

## Results

### Patient characteristics

Patient characteristics were collected through a prospectively maintained database, updated at every patient’s visit in UHF. Baseline characteristics of the proportion of the ACLF-I cohort described in this paper reflect an advanced cirrhosis population, with a median age of 58 years, and a model-of-end-stage-liver-disease (MELD) score of 17 demonstrating the expected gradients in hepatic dysfunction and organ failure burden. The most common etiology was alcoholism (54.5%). Two patients underwent liver transplantation prior to NCR-DRGN detection. Decompensation at admission presented itself with ascites (45.5%) and spontaneous bacterial peritonitis (4.5%), hepatic encephalopathy (13.6%), or gastrointestinal bleeding (9.1%). All patients were screened on admission as they belong to a defined risk group to be colonized with NCR-DRGN. Characteristics of NCR-DRGN-positive patients are depicted in Table 1.

**TABLE 1:** Patient characteristics of NCR-DRGN -positive patients included in this study.

Parameter	Total *n* = 22
Age median (interquartile range)	58.0 (50.0–63.75)
Male sex *n* (%)	*n* = 16 (72.7%)
Etiology (*n*, %)	*n* = 22
Alcoholic	*n* = 12 (54.5%)
Hepatitis B	*n* = 2 (9.1%)
Hepatitis C	*n* = 1 (4.5%)
Hepatitis B and C	*n* = 1 (4.5%)
MASLD/MASH	*n* = 3 (13.6%)
Other	*n* = 3 (13.6%)
Decompensation at admission (*n*, %)	*n* = 16/22
Ascites	*n* = 10 (45.5%)
With SBP	*n* = 1 (4.5%)
Hepatic encephalopathy	*n* = 3 (13.6%)
Gastrointestinal bleeding	*n* = 2 (9.1%)
MELD median (interquartile range)	17.0 (13.25–24.25)
Colonization with NCR-DRGN (*n*, %)	*n* = 18 (81.8%)
Infection with NCR-DRGN (*n*, %)	*n* = 4 (18.2%)
LT prior to NCR-DRGN detection	*n* = 2 (9.1%)

MASLD, metabolic dysfunction-associated steatotic liver disease; MASH, metabolic dysfunction-associated steatohepatitis; SBP, spontaneous bacterial peritonitis; MELD, model of end stage liver disease; LT, liver transplantation. Data represented as median (IQR) or n (%) as appropriate.

### Prevalence and antibiotic resistance profiles of NCR-DRGN isolates

**FIGURE 1:**
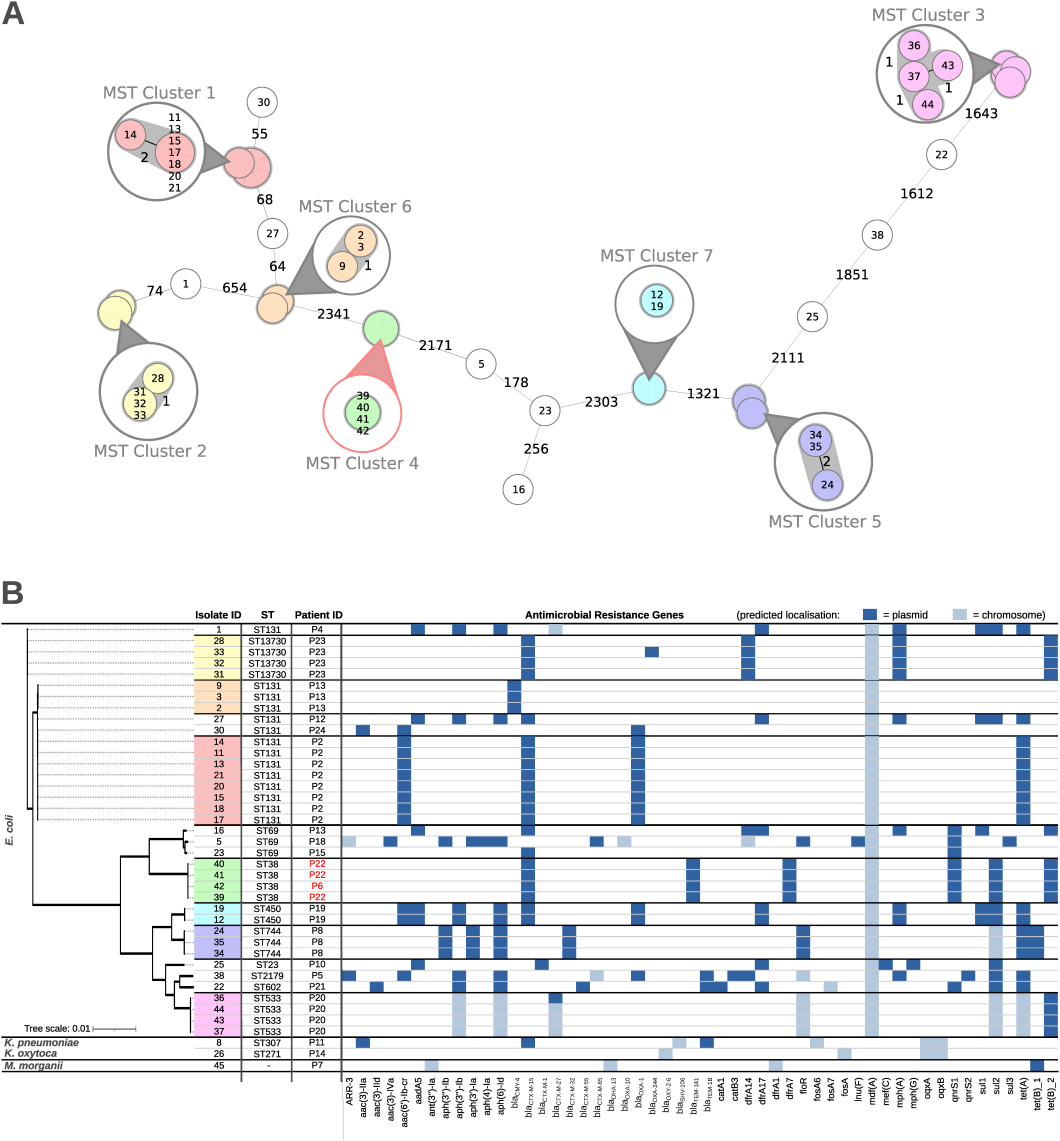
Bioinformatic analysis of *n* = 40 bacterial isolates. A minimal spanning tree (MST) for *E. coli* isolates is given in **(A)**. Detailed depictions are drawn in circles for each cluster. The cluster with isolates originating from two patients is colored in red instead of gray. A summary of all *in silico* findings is provided in **(B)** for each isolate. Isolate identifiers are marked with colored boxes according to their respective MST cluster, and patient numbers are marked in red if there is more than one patient in the MST cluster. Found antimicrobial resistance genes are colored based on predicted origin (plasmid in dark blue, chromosome in light blue).

Over a 32-months period, 22 out of 182 (12.1%) screened patients tested positive for at least one NCR-DRGN organism. A total of 44 distinct NCR-DRGN isolates were identified, including *Escherichia coli* (*n* = 40), *Klebsiella pneumoniae* (*n* = 2), *Klebsiella oxytoca* (*n* = 1), and *Morganella morganii* (*n* = 1) (see Table 2). Three *E. coli* isolates and one *K. pneumoniae* isolate were excluded from sequencing analysis because they could not be regrown from the in-house isolate collection. All isolates showed phenotypic resistance against third-generation cephalosporins but not against carbapenems. One particular *E. coli* isolate (isolate 33) was initially classified as NCR-DRGN having an ESBL phenotype, while WGS performed later in this study revealed the presence of *bla*_CTX–M–15_ and *bla*_OXA–244_ gene (Figure 1 and Table 3).

**TABLE 2:** Bacterial isolate identification and antibiotic resistance profile (sequenced isolates: *n* = 40).

Isolate number	ST	Patient no.	Sampling site	Species
25	23	P10	Rectal	*E. coli*
41	38	P22	Throat	*E. coli*
39	38	P22	Rectal	*E. coli*
40	38	P22	Rectal	*E. coli*
42	38	P6	Rectal	*E. coli*
16	69	P13	Skin	*E. coli*
23	69	P15	Rectal	*E. coli*
5	69	P18	Rectal	*E. coli*
27	131	P12	Urine	*E. coli*
9	131	P13	Rectal	*E. coli*
2	131	P13	Rectal	*E. coli*
3	131	P13	Rectal	*E. coli*
14	131	P2	Nose/throat	*E. coli*
11	131	P2	Throat	*E. coli*
18	131	P2	Rectal	*E. coli*
20	131	P2	Rectal	*E. coli*
21	131	P2	Rectal	*E. coli*
13	131	P2	Rectal	*E. coli*
15	131	P2	Rectal	*E. coli*
17	131	P2	Rectal	*E. coli*
30	131	P24	Rectal	*E. coli*
1	131	P4	Rectal	*E. coli*
12	450	P19	Throat	*E. coli*
19	450	P19	Rectal	*E. coli*
43	533	P20	Rectal	*E. coli*
36	533	P20	Rectal	*E. coli*
37	533	P20	Rectal	*E. coli*
44	533	P20	Rectal	*E. coli*
22	602	P21	Rectal	*E. coli*
34	744	P8	Rectal	*E. coli*
24	744	P8	Rectal	*E. coli*
35	744	P8	Rectal	*E. coli*
38	2,179	P5	Rectal	*E. coli*
33	13,730	P23	Skin	*E. coli*
28	13,730	P23	Rectal	*E. coli*
31	13,730	P23	Rectal	*E. coli*
32	13,730	P23	Rectal	*E. coli*
6*	–	P1	Rectal	*E. coli*
7*	–	P1	Rectal	*K. pneumoniae*
4*	–	P16	Rectal	*E. coli*
10*	–	P3	Rectal	*E. coli*
26	271	P14	Rectal	*K. oxytoca*
8	307	P11	Skin	*K. pneumoniae*
45	–	P7	Urine	*M. morganii*

Sample-ID, unique identifier for each sample; ST, sequence type of the bacterial strain; Patient no., patient number; Species: bacterial species present in each sample. Bacterial isolates are sorted according to their respective sequence type (ST).

*Isolate was not available for whole-genome sequencing.

**TABLE 3:** Comparative analysis of resistance to β-lactam antibiotics (phenotypic versus genotypic).

Patient #	Isolate #	Species	AMS	TZP	CTX	CAZ	IMI	MEM	*bla*-genes
P1	6	*E. coli*	R	I	R	R	S	S	Not sequenced
P1	7	*K. pneumoniae*	R	I	R	R	S	S	Not sequenced
P2	11	*E. coli*	R	I	R	R	S	S	CTX-M-15, OXA-1
P2	13	*E. coli*	R	I	R	R	S	S	CTX-M-15, OXA-1
P2	14	*E. coli*	R	I	R	R	S	S	CTX-M-15, OXA-1
P2	15	*E. coli*	R	R	R	R	S	S	CTX-M-15, OXA-1
P2	17	*E. coli*	R	R	R	R	S	S	CTX-M-15, OXA-1
P2	18	*E. coli*	R	R	R	R	S	S	CTX-M-15, OXA-1
P2	20	*E. coli*	R	R	R	R	S	S	CTX-M-15, OXA-1
P2	21	*E. coli*	R	R	R	R	S	S	CTX-M-15, OXA-1
P3	10	*E. coli*	R	I	R	R	S	S	Not sequenced
P4	1	*E. coli*	R	I	R	R	S	S	CTX-M-15, CTX-M-27
P5	38	*E. coli*	R	I	R	R	S	S	CTX-M-65, OXA-1, TEM-1B
P6	42	*E. coli*	R	I	R	R	S	S	CTX-M-15, TEM-141
P7	45	*M. morganii*	R	R	R	R	I	S	DHA-13
P8	24	*E. coli*	R	I	R	R	S	S	CTX-M-32
P8	34	*E. coli*	R	I	R	R	S	S	CTX-M-32
P8	35	*E. coli*	R	I	R	R	S	S	CTX-M-32
P10	25	*E. coli*	R	I	R	R	S	S	CTX-M-1
P11	8	*K. pneumoniae*	R	R	R	R	S	S	CTX-M-15, SHV-106, TEM-1B
P12	27	*E. coli*	R	I	R	R	S	S	CTX-M-15
P13	2	*E. coli*	R	R	R	R	S	S	CMY-4
P13	3	*E. coli*	R	I	R	R	S	S	CMY-4
P13	9	*E. coli*	R	R	R	R	S	S	CMY-4
P13	16	*E. coli*	R	I	R	R	S	S	CTX-M-15
P14	26	*K. oxytoca*	R	R	R	R	S	S	OXY-2-6
P15	23	*E. coli*	R	I	R	R	S	S	CTX-M-15
P16	4	*E. coli*	R	I	R	R	S	S	Not sequenced
P18	5	*E. coli*	R	I	R	R	S	S	CTX-M-65, OXA-10
P19	12	*E. coli*	R	I	R	R	S	S	CTX-M-15, OXA-1
P19	19	*E. coli*	R	I	R	R	S	S	CTX-M-15, OXA-1
P20	36	*E. coli*	R	I	R	R	S	S	CTX-M-27
P20	37	*E. coli*	R	I	R	R	S	S	CTX-M-27
P20	43	*E. coli*	R	I	R	R	S	S	CTX-M-27
P20	44	*E. coli*	R	I	R	R	S	S	CTX-M-27
P21	22	*E. coli*	R	I	R	R	S	S	CTX-M-55, TEM-1B
P22	39	*E. coli*	R	I	R	R	S	S	CTX-M-15, TEM-141
P22	40	*E. coli*	R	I	R	R	S	S	CTX-M-15, TEM-141
P22	41	*E. coli*	R	I	R	R	S	S	CTX-M-15, TEM-141
P23	28	*E. coli*	R	I	R	R	S	S	CTX-M-15
P23	31	*E. coli*	R	I	R	R	S	S	CTX-M-15
P23	32	*E. coli*	R	I	R	R	S	S	CTX-M-15
P23	33	*E. coli*	R	I	R	R	S	S	CTX-M-15, OXA-244
P24	30	*E. coli*	R	I	R	R	S	S	OXA-1

AMS, ampicillin/sulbactam; TZP, piperacillin/tazobactam; CTX, cefotaxime; CAZ, ceftazidim; IMI, imipenem; MEM, meropenem; R, resistant; S, susceptible.

### Sequence typing and species distribution

Bioinformatics analysis of genome assembly yielded an average N50 value of 200 kb, with most genomes comprising fewer than 150 contigs. The majority of isolates (37 isolates obtained from 16 patients) were classified as *E. coli*. Distinct sequence types (STs) were observed among patients, with ST131 present in multiple patients (P2, P4, P12, P13, and P24). In addition, ST69 was identified in three (P13, P15, P18) and ST38 in two patients (P6, P22). Furthermore, three non-*E. coli* species were identified: (i) M. morganii (not typeable, P7), (ii) K. oxytoca (ST271, P14), and (iii) *K. pneumoniae* (ST307; P11) (Figure 1 and Table 2). The majority (*n* = 7/10) of *E. coli* STs were represented by only one particular isolate or multiple isolates (“copy strains”) from the same patient (Figure 1). Among the remaining STs, ST131 was most frequently detected in five patients, and, when including ST13730 (belonging to the clonal complex CC131), even in six patients. This variability in STs suggests multiple independent sources of bacterial introduction (except for P6 and P22) rather than inter-patient transmission.

### Antibiotic resistance gene analysis

A number of 15 different β-lactam-resistance genes were detected. One isolate from one patient (isolate 33, P23) carried a *bla*_OXA–244_ gene, indicating impaired carbapenem susceptibility. All remaining isolates lacked the presence of carbapenemase genes. For all other isolates, no differences between the phenotypic or genotypic resistance profiles were detected (see Table 3).

In total, 12 ESBL-genes from the *bla*_CTX–M_-, *bla*_OXA_-, *bla*_OXY_-, *bla*_SHV_- and *bla*_TEM_-type, two AmpC genes (*bla*_CMY–4_, *bla*_DHA–13_) and one carbapenemase gene (*bla*_OXA–244_) were found, mostly predicted as plasmid-borne, indicating the possibility of intra- or interspecies transmission. Additionally, the following resistance genes were found spread among all isolates: 11 different genes for aminoglycoside-resistance (*aac*, *ant*, *aad*, *aph*), one for rifamycine resistance (*arr*), three for chloramphenicol resistance (*cat*, *flo*), four for diaminopyrimidine resistance (*dfr*), three for fosfomycin resistance (*fos*), one for lincosamide resistance (*Inu*), three for tetracycline resistance (*tet*), two for macrolides resistance (*mph*), two for fluoroquinolone resistance (*qnr*), three for sulphonamide resistance (*sul*) and the rest represented by other efflux pumps (*mef*, *oqx*) with one (*mdf*) coding for a multidrug resistance protein conferring resistance against rifampicin, tetracyclines, chloramphenicol, erythromycin, aminoglycosides and fluoroquinolones as well as benzalkonium, a quaternary ammonium compound disinfectant. Figure 1B provides an overview of the detected resistance genes associated with the isolates and their genomic localization (plasmid or chromosomal).

### Core genome multilocus sequence typing (cgMLST) analysis

**FIGURE 2:**
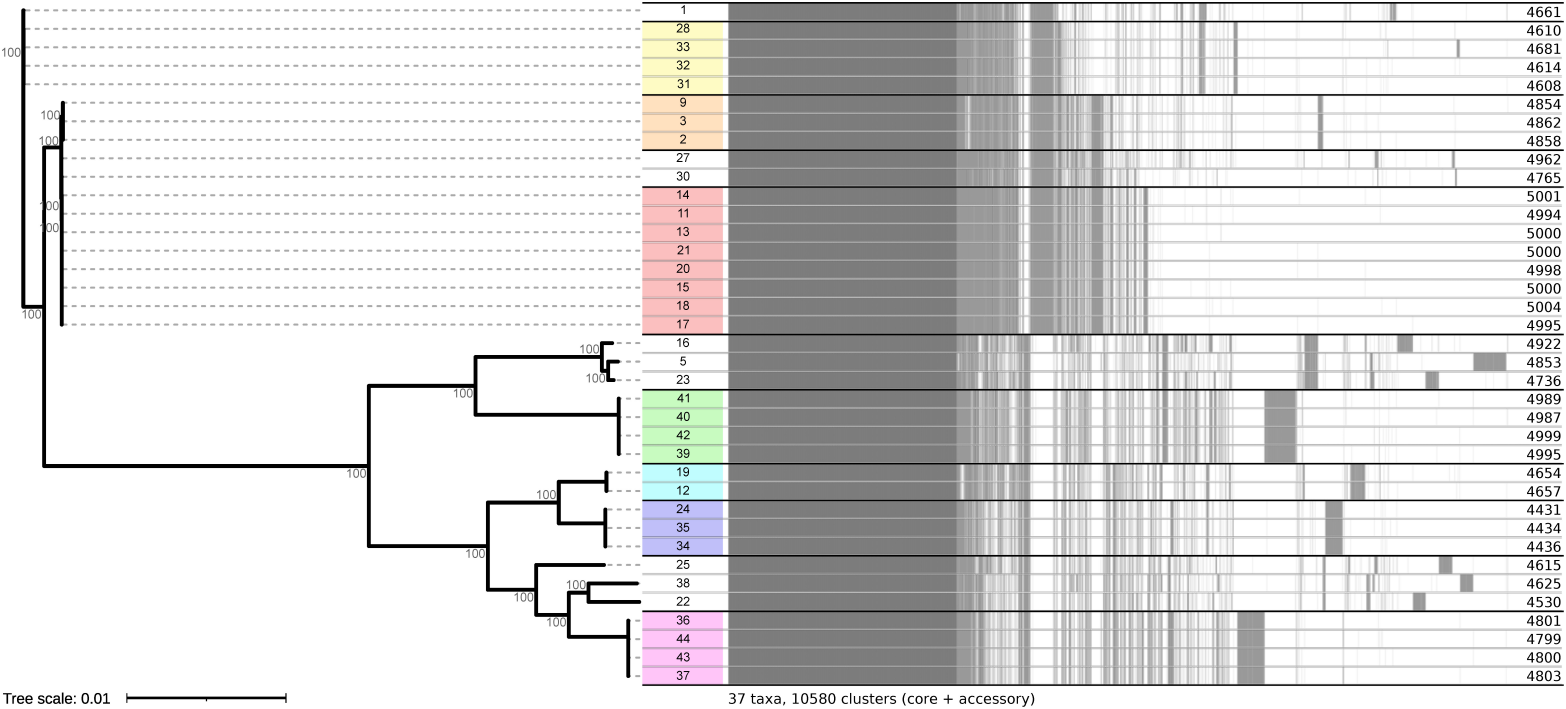
Pangenome plot of the 37 *E. coli* isolates included in this study. A phylogenetic tree based on the sequences of core genes is provided on the left side including bootstrap values for branches. A presence-absence matrix for all identified genes is drawn on the right, where a homologue in the respective isolate is indicated by a gray line. Isolate identifiers (in the middle) were highlighted based on their respective cluster in the cgMLST analysis. Overall, the pangenome analysis fully supports the cgMLST clusters (Figure 1 and Table 2).

An allele difference of ≤ 10 was defined as clustering threshold indicating closely related isolates. The observed allele differences for isolates within clusters range between 0 and 2, while the closest intercluster allele difference was 55. Clusters were further supported by pangenome analysis (Figure 2). The cgMLST analysis highlighted the presence of multiple STs among the *E. coli* isolates (Figure 1). While certain STs were unique to individual patients, others were shared among multiple patients. Notably, although MLST identified ST131 in 14 isolates originating from five patients (Table 1), cgMLST analysis demonstrated significant genetic diversity within these isolates, indicating multiple independent introductions rather than direct transmission (Figure 1). Again, ST38 was observed in both P6 and P22, forming a distinct cluster in the minimum spanning tree (MST) which suggests transmission of NCR-DRGN bacteria between these two ACLD patients (no allele differences nor core gene single nucleotide variants in isolates 39–42). These findings underscore the genetic variability among isolates and the potential transmission dynamics within the study population.

## Discussion

In this study, we determined that (i) approximately 12% (*n* = 22/182) of ACLD patients are colonized or infected with NCR-DRGN, (ii) 97.5% (*n* = 39/40) of the sequenced bacteria were NCR-DRGN without carbapenemase genes, while 2.5% (*n* = 1/40) harbored a carbapenemase gene (*bla*_OXA–244_), and (iii) *E. coli* was the predominant multidrug-resistant pathogen (*n* = 40/44; 90.9%) among ACLD patients. A close phylogenetic relationship was observed only between two *E. coli* strains from two patients, although the mode of inter-patient transmission of this strain remains unclear. Thus, our study provides a comprehensive assessment of NCR-DRGN colonization and transmission dynamics in ACLD patients, integrating patient chart reviews, conventional microbiological techniques, and WGS. With 12.1% of ACLD patients colonized by NCR-DRGN bacteria, our cohort exhibits a colonization burden lower than comparable data for European cirrhosis patients ranging from 13.3% to 29.9% (Pouriki et al., 2018; Ferstl et al., 2021; Kremer et al., 2022). Recent data from India highlight global variability, reporting a prevalence of 55.4% on admission comparable to our setting (Verma et al., 2023). Looking at similar high-risk patient cohorts, MDRGN colonization is comparable in German hemodialysis patients with 10.4% (Wendt et al., 2020) but generally higher in patients with hematological malignancies, ranging worldwide between 21.7% and 28% (Rogacheva et al., 2022; Luo et al., 2024; Gallardo-Pizarro et al., 2025). In general hospital settings, the admission prevalence of NCR-DRGN bacteria has been reported at approximately 10% in a German tertiary care center (Boldt et al., 2018). These findings imply that the risk of NCR-DRGN colonization in our ACLD patient cohort did not differ significantly from that of the general hospital patient population.

Recent data have highlighted that NCR-DRGN *E. coli* pose a significant health risk, with *E. coli* resistant to β-lactam/β-lactamase inhibitors contributing substantially to overall AMR-attributable mortality (Meštrović et al., 2025). This finding is consistent with our own observation that approximately 91% of the isolated strains belong to the *E. coli* species. Clearly, we did not observe a clonal outbreak or similar scenario. All strains belonged to different STs or genetic clusters, which largely excludes the possibility of patient-to-patient transmission. Genome analyses revealed high genetic variability, even within identical STs, suggesting a low likelihood of transmission within our cohort. Importantly, in 39 out of 40 sequenced bacterial isolates (97.5%), at least one resistance gene was localized on plasmids (Figure 1B) indicating the possibility for intra- or interspecies transmission of such resistance plasmids as shown before (Göttig et al., 2015).

One potential transmission event involving ST38 between patients P6 and P22 was identified but could not be clarified through temporo-spatial analysis, as the patients were never simultaneously in our hospital, indicating most likely a way of indirect pathogen transmission, e.g., via medical devices, wastewater from hospital sanitation systems, or inadequate hand disinfection by medical staff. Nevertheless, documented transmissions of CR-MDRGN in both outpatient clinics (Klein et al., 2021) and inpatient settings (Chapuis et al., 2016; Weber et al., 2019), including ST38 harboring *bla*_OXA–244_ (Kremer et al., 2020) underscore the clinical importance of robust screening, strict hygiene protocols, and prompt isolation of colonized patients to avoid “silent” outbreak scenarios in this vulnerable patient cohort.

Genomic analyses further underscore the complexity of NCR-DRGN dynamics. Sequence typing revealed diverse STs without dominant clustering, although high-risk STs such as ST131, ST69, and ST38 were prevalent. These STs are globally associated with extraintestinal infections (Manges et al., 2019), with ST131 being recognized as a pandemic MDR strain often carrying *bla*_CTX–M–15_ (Whitmer et al., 2019). This, along with the potential for carbapenem resistance (CR) evolution, highlights an elevated risk for ACLD patients. Interestingly, one bacterial isolate (*K. pneumoniae*, isolate 8) was identified to belong to the ST307 which has been described to harbor so-called “hypervirulent” *K. pneumoniae* (Heiden et al., 2020). Own genomic analysis revealed, however, that this strain did not harbor the hypervirulence genes *rmpA*, *rmpA2* nor *iucA-D* (Russo and Marr, 2019).

Adding to the complexity, antibiotic resistance gene analysis in our cohort revealed a non-detected carbapenemase-producing Gram-negative *E. coli* (isolate 33) with phenotypic carbapenem-susceptibility. This underscores the challenges faced by clinical microbiology laboratories, particularly the need for rigorous detection methods as recommended by EUCAST and the German “National Antibiotic Sensitivity Testing Committee” (NAK) criteria. Phenotypic test sensitivities for detecting CR-MDRGN vary widely, ranging from 55% to 89% across all Gram-negative bacteria and 51% to 82% for *Enterobacterales* (Kamel et al., 2022). Reduced hydrolytic activity against meropenem, particularly in OXA-48-like carbapenemases, such as *bla*_OXA–244_ identified in our case, can further impair screening sensitivity in agar diffusion testing (Huang et al., 2014). Notably, *bla*_OXA–244_ can yield carbapenem MICs within susceptible or intermediate ranges (here: 1 mg/l), and even phenotypic cephalosporin susceptibility (Hoyos-Mallecot et al., 2017; Rima et al., 2021).

The potential for resistance evolution within NCR-DRGN isolates presents an additional concern. Several β-lactamase genes identified in our cohort possess the capacity to evolve into carbapenemase producers. For example, *bla*_CTX–M–33_, a point mutation variant of *bla*_CTX–M–15_, confers meropenem hydrolysis (Poirel et al., 2019), while *bla*_OXA–655_ and *bla*_OXA–656_—both derivatives of *bla*_OXA–10_—may impair carbapenem susceptibility (Kotsakis et al., 2019). CR due to combined mechanisms was observed in *bla*_CMY–4_ carrying bacteria with porin loss (Stapleton et al., 1999). These findings reinforce the risk of CR emergence under selective pressure (Sommer et al., 2009; Meletis et al., 2014; Iredell et al., 2016), emphasizing the importance of stringent infection control and antimicrobial stewardship, particularly in vulnerable patient groups.

Although this study provides insights into NCR-DRGN transmission in ACLD patients, its single-center design may limit the generalizability of the findings and may overlook undocumented interactions. As we did not observe an epidemiological outbreak within the group of ACLD patients, we can conclude that the standard hygiene measures in our hospital are sufficient to prevent pathogen transmission. It is important to note that no special or strict hygiene protocols have been established for this patient cohort (e.g., no strict isolation of patients, visitor restrictions, or similar measures). Instead, we rely on basic hygiene procedures (hand disinfection, surface cleaning, etc.), in accordance with the national hygiene recommendations dating from 2012 (Bundesgesundheitsblatt - Gesundheitsforschung - Gesundheitsschutz, 2012). These data demonstrate that standard hospital hygiene practices might prevent pathogen spread, which is a significant observation in the context of rising bacterial resistance.

## Data Availability

The datasets presented in this study can be found in online repositories. The names of the repository/repositories and accession number(s) can be found below: https://www.ncbi.nlm.nih.gov/genbank/, PRJNA1219355.

## Footnotes

1
https://www.chromagar.com/wp-content/uploads/2021/11/LF-EXT-019-ES-V7.1.pdf

2
https://enterobase.warwick.ac.uk/species/index/ecoli

3
https://raw.githubusercontent.com/tseemann/nullarbor/master/bin/roary2svg.pl

4
https://klif.uu.nl/rfplasmid/
